# Malnutrition in a Sample of Community-Dwelling People with Parkinson’s Disease

**DOI:** 10.1371/journal.pone.0053290

**Published:** 2013-01-09

**Authors:** Jamie M. Sheard, Susan Ash, George D. Mellick, Peter A. Silburn, Graham K. Kerr

**Affiliations:** 1 School of Exercise and Nutrition Sciences, Queensland University of Technology, Brisbane, Queensland, Australia; 2 Movement Neuroscience Program, Institute of Health and Biomedical Innovation, Queensland University of Technology, Brisbane, Queensland, Australia; 3 Australia Eskitis Institute for Cell and Molecular Therapies, Griffith University, Brisbane, Queensland, Australia; 4 University of Queensland Centre for Clinical Research, Brisbane, Queensland, Australia; Oslo University Hospital, Norway

## Abstract

**Objective:**

Malnutrition results in poor health outcomes, and people with Parkinson’s disease may be more at risk of malnutrition. However, the prevalence of malnutrition in Parkinson’s disease is not yet well defined. The aim of this study is to provide an estimate of the extent of malnutrition in community-dwelling people with Parkinson’s disease.

**Methods:**

This is a cross-sectional study of people with Parkinson’s disease residing within a 2 hour driving radius of Brisbane, Australia. The Subjective Global Assessment (SGA) and scored Patient Generated Subjective Global Assessment (PG-SGA) were used to assess nutritional status. Body weight, standing or knee height, mid-arm circumference and waist circumference were measured.

**Results:**

Nineteen (15%) of the participants were moderately malnourished (SGA-B). The median PG-SGA score of the SGA-B group was 8 (4–15), significantly higher than the SGA-A group, *U* = 1860.5, *p*<.05. The symptoms most influencing intake were loss of appetite, constipation, early satiety and problems swallowing.

**Conclusions:**

As with other populations, malnutrition remains under-recognised and undiagnosed in people with Parkinson’s disease. Regular screening of nutritional status in people with Parkinson’s disease by health professionals with whom they have regular contact should occur to identify those who may benefit from further nutrition assessment and intervention.

## Introduction

The frequency of malnutrition, defined as protein-energy undernutrition, in people with Parkinson’s disease (PWP) is reported to be 0–2% using the Mini-Nutritional Assessment (MNA) [1], [2] with a further 20−34% at risk of malnutrition [1], [2]. Similarly, Jaafar, et al [3] reported that 24% of community-dwelling PWP were at medium to high risk of malnutrition using the Malnutrition Universal Screening Tool (MUST). This reported frequency of malnutrition is lower than may be expected given that significant amounts of weight loss have previously been reported in PWP [4]–[6]. Due to limitations such as sampling bias towards more mobile participants without cognitive impairment [7], the frequency of malnutrition in PWP reported to date is likely to be under-reported.

Malnutrition results from inadequate calories, protein or other nutrients that are required for maintenance and repair of body tissues. Appropriate identification of and interventions for malnutrition should be a priority in order to prevent or delay the associated poor outcomes such as decreased quality of life, longer recovery from illness, higher likelihood of falls, increased risk for osteoporosis and increased risk of hospitalisation and admittance to aged care facilities [8]–[13]. The etiology of malnutrition is multi-factorial, and nutrition assessment tools, such as the MNA and the Subjective Global Assessment (SGA), incorporate anthropometry, weight history, dietary intake and physical signs of malnutrition in the criteria for a diagnosis of malnutrition. The scored Patient Generated Subjective Global Assessment (PG-SGA), which incorporates the SGA, also includes assessment of nutrition impact symptoms, such as poor appetite, nausea, constipation, problems swallowing, early satiety and depression. These symptoms commonly occur in Parkinson’s disease and can provide information about appropriate intervention strategies. The recording of nutrition impact symptoms also allows for monitoring and evaluation of the effectiveness of both medical and nutritional interventions. Therefore, the use of the SGA and the scored PG-SGA in this population appears to be the most appropriate choice particularly as the SGA is a valid tool for use in the community setting [14].

The current study aims to use a nutrition assessment tool, the SGA to assess nutritional status to better estimate the frequency of malnutrition in community-dwelling people with Parkinson’s disease in Australia.

## Subjects and Methods

**Figure 1:**
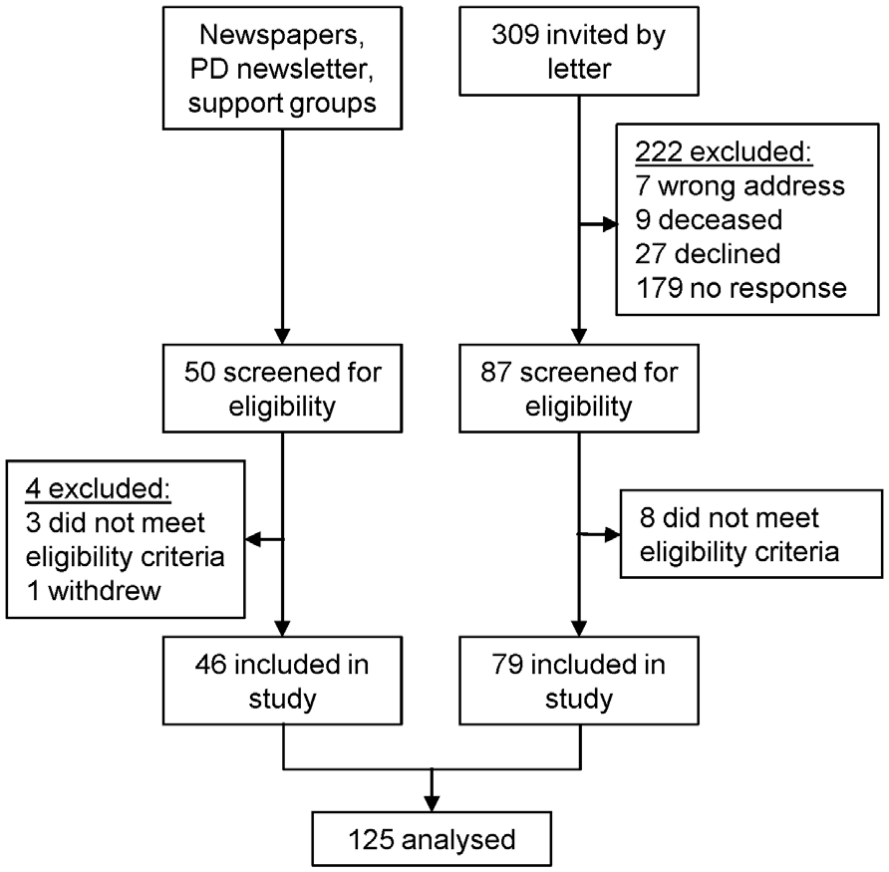
Recruitment process.

Community-dwelling people with PD, aged >18 years were recruited using a variety of methods between February and August 2011: invitations to previous research participants who had expressed an interest in involvement in future studies, appearances at community Parkinson’s disease support groups, advertisement in Queensland-wide Parkinson’s disease organisation newsletter and media coverage in local and state newspapers. Participants were excluded if they resided in an assisted living facility (Figure 1). Geographical location was limited to areas within ∼2 hour driving distance from Brisbane, Queensland, Australia.

### Ethics Statement

Informed written consent was obtained as per protocol approved by the Queensland University of Technology Human Research Ethics Committee which also approved the study (#1000001150).

Data collection was completed either at Queensland University of Technology Institute of Health and Biomedical Innovation (n = 15) or at participants’ homes (n = 110). During the data collection visit, disease duration was obtained from the participant and/or their spouse.

Nutritional status was measured by a dietitian using the Subjective Global Assessment (SGA) [15]. Nutrition assessment using the SGA is based on a medical history (recent changes in weight, dietary intake, gastrointestinal symptoms, functional capacity and disease status) and a physical examination of fat stores, muscle status and fluid status. The assessment results in a categorisation of nutrition status: SGA-A (well nourished), SGA-B (moderately malnourished) or SGA-C (severely malnourished). The SGA has been used extensively for nutrition assessment and is validated for use in many different patient groups [16]. The scored Patient-Generated Subjective Global Assessment (PG-SGA) was also completed, which uses the same global nutrition assessment rating as the SGA but also provides a total score from four worksheets with a higher score indicating poorer nutritional status [17].

PG-SGA Worksheet 1, which is completed by the participant, provides a score for recent changes in weight, food intake, nutrition impact symptoms and functional capacity. Worksheet 2 provides a score for medical conditions and age. Worksheet 3 provides a score for components of metabolic stress, and finally Worksheet 4 consists of the physical examination score. Worksheets 2, 3 and 4 are completed by the assessor. The scores from the worksheets are totaled to provide the PG-SGA score.

Anthropometric and body composition data were collected while fasting. Body weight was measured to the nearest 0.1 kg (Tanita HD-316, Japan) in light clothing, without shoes. For those participants able to stand independently, height was measured to the nearest 0.5 cm, without shoes. For those participants unable to stand independently or with marked stooped posture, knee height was used to estimate height. Knee height was measured to the nearest 0.1 cm using a knee caliper on the left leg with the knee and ankle at a 90° angle. The equation used for men was height (cm) = 78.31+(1.94×knee height) − (0.14×age), and the equation used for women was height (cm) = 82.21+(1.85×knee height)−(0.21×age). [18] BMI was calculated (body weight(kg)/(height in metres)^2^). Mid-arm circumference (MAC) was measured to the nearest 0.1 cm at the mid-point between the acromion and the olecranon process on the non-dominant arm [19]. Calf circumference was measured to the nearest 0.1 cm at the widest part of the calf with the knee at 90° [20]. Waist circumference was measured at the mid-point between the iliac crest and the lower rib [19]. Some participants presented with visible peripheral oedema but no adjustments were made to the weight or calf circumference measurements to account for this.

The Hoehn and Yahr (H&Y) scale, which is a five point scale (1–5) with a higher rating on the scale indicating a greater amount of disability and impairment, was also measured. The H&Y classifications were split into 2 groups (less severe PD H&Y 0–3, severe PD H&Y 4–5).

Statistical analysis was completed using SPSS Version 19 (SPSS Inc., Chicago, IL, USA). Missing data were not included in the analysis. Variables were compared between SGA-A and SGA-B classifications only as no one was classified as SGA-C. The only variable of interest that was normally distributed in the SGA-A group was height. The only variable that was normally distributed for both males and females was also height. Therefore, non-parametric Mann-Whitney U tests were conducted to compare the scores between the groups. Although within the SGA-B group, the variables age, height, weight, MAC, calf circumference, PD duration and PG-SGA score were all normally distributed for both males and females, non-parametric tests were all conducted for consistency in reporting. Pearson’s Chi square tests were used to evaluate the differences in categorical variables. Statistical significance was set at p<0.05.

## Results

One hundred twenty five community dwelling adults over the age of 18 years (74 M, 51 F) agreed to participate. Characteristics of those who did not participate were not known. Of the 27 that provided a reason for not participating, four replied that they were too ill, one stated immobility as the reason, two replied that participation was too stressful, seven were not interested in the research project, and the remaining 13 were not available or too busy to participate. Median age of the participants was 70.0 years (range: 35–92), and 72.8% (n = 91) were over the age of 65 years. Median age at diagnosis was 63.0 years (range: 28.0–84.0), and self-reported median disease duration was 6.0 years (range: 0–31). The median H&Y score was 2 with 92.8% of participants in the less severe category and 7.2% in the more severe category. Median BMI was 25.1 kg/m^2^ (range: 17.0–49.5) with 4 participants (3.2%) falling within the World Health Organisation (WHO) underweight classification (<18.5 kg/m^2^). One participant could not recall when diagnosis had occurred. Five participants did not have a waist circumference measurement, and one participant did not have a calf circumference measurement.

Nineteen (15.2%) participants were moderately malnourished (SGA-B) while none were severely malnourished (SGA-C). In the SGA-B category, 3 (15.8%) of the participants were <65 years old. Of the 125 participants, 54 (43%) (28 M, 26 F) reported previous unintentional weight loss at some point following diagnosis. The well-nourished participants had significantly higher anthropometric measures (BMI, *U* = 367.0, *z = *−4.40, *p* = .000; MAC, *U* = 482.5, *z = *−*3.61, p* = .000; calf circumference, *U* = 355.5, *z = *−4.46, *p* = .000; and waist circumference, *U* = 410.5, *z = *−3.47, *p* = .001) than the malnourished participants. Significantly lower PG-SGA scores, *U* = 1860.5,*z = *5.94,*p* = .000 were found for the well-nourished participants (Table 1). This difference in total score was determined by the significant differences in the scores for Worksheet 1 (SGA A Mdn = 1, SGA B Mdn = 6), *U* = 1811.5, *z = *5.64, *p* = .000, and Worksheet 4 (SGA A Mdn = 0, SGA B Mdn = 1), *U* = 1783.0, *z = *6.37, *p* = .000. The scores for Worksheets 2 and 3 were not significantly different between the two groups. The score for Worksheet 3 was 0 for all participants.

**Table 1:** Characteristics of the participants compared between nutritional status categories using Mann-Whitney analysis for continuous variables and Chi square analysis for categorical variables and reported as median (range).

	SGA Classification	
	A (Well-nourished) (n = 116)	B (Moderately malnourished) (n = 19)
Age (years)	69.0 (35.0–92.0)	74.0 (61.0–87.0)
Height (cm)	167.25 (148.0–186.0)	164.8 (140.5–174.0)
Weight (kg)	73.2 (46.5–145.0)*	55.4 (40.7–100.6)*
BMI (kg/m^2^)	25.9 (17.0–49.5)*	20.0 (17.7–33.2)*
MAC (cm)	29.1 (21.0–43.0)*	25.5 (21.0–35.0)*
Waist circumference (cm)	95.5 (67.5–149.5)*	82.5 (65.5–116.0)*
Calf Circumference (cm)	36.5 (31.0–48.5)*	32.5 (28.0–39.0)*
PG-SGA score	2 (0–15)*	8 (4–15)*

* Statistically significant difference between SGA classifications (p<0.05).

† Reported as frequencies (percentages). Abbreviations: BMI = body mass index, MAC = mid-arm circumference, PD = Parkinson’s disease, PG-SGA = Patient-Generated Subjective Global Assessment, SGA = Subjective Global Assessment.

‘No appetite or not feeling like eating’ was the symptom reported most frequently on the PG-SGA for both SGA-A (13%) and SGA-B (53%) groups, *X* ^2^(1, n = 125) = 14.91,*p*<.05 (Table 2). In addition to ‘no appetite’, the differences between groups reached significance for constipation, *X* ^2^(1, n = 125) = 8.11, *p*<.05, diarrhea, *X* ^2^(1, n = 125) = 6.32, *p*<.05, problems swallowing, *X* ^2^(1, n = 125) = 7.08, *p*<.05, and feel full quickly, *X* ^2^(1, n = 125) = 16.81, *p*<.05.

**Table 2:** Nutrition impact symptoms as reported on the PG-SGA compared between SGA groups using Chi-square analysis.

	SGA Classification	
	A (Well-nourished) (n = 116)	B (Moderately malnourished) (n = 19)
No appetite, just did not feel like eating	15 (13%)*	10 (53%)*
Nausea	4 (3%)	1 (5%)
Vomiting	1 (1%)	0
Constipation	2 (2%)*	3 (16%)*
Diarrhea	1 (1%)*	1 (5%)*
Mouth sores	2 (2%)	0
Dry mouth	6 (5%)	1 (5%)
Things taste funny or have no taste	9 (8%)	4 (21%)
Smells bother me	1 (1%)	0
Problems swallowing	10 (9%)*	6 (32%)*
Feel full quickly	7 (6%)*	8 (42%)*
Pain	2 (2%)	1 (5%)
Other	5 (4%)	0

* Statistically significant difference between SGA classifications (p<0.05). Abbreviations: PG-SGA = Patient-Generated Subjective Global Assessment, SGA = Subjective Global Assessment.

The PG-SGA score for 53% of the SGA B participants placed them into the triage category requiring intervention by a dietitian (scores 4–8), and the remaining 47% fell into the triage category indicating a critical need for improved symptom management and/or nutrient intervention options (scores ≥9). Comparatively, 21% and 9% of the SGA A participants were placed into these categories, respectively. The remaining SGA A participants were evenly split between the category requiring no intervention (scores 0–1) and the category requiring patient and family education (scores 2–3). This categorisation was significantly different between the groups, *X* ^2^ (3, n = 125) = 34.91, *p*<.05.

Of the 19 malnourished participants, 9 were female (47%). A similar proportion of females (18%) were malnourished than males (14%) as assessed using Chi square analysis. A greater proportion of females (50%) also experienced unintentional weight loss when compared to males (38%). Females who were malnourished had significantly lower BMIs, *U* = 71.5, *z = *2.17, *p* = .028, smaller mid-arm circumferences, *U* = 80.0, *z = *2.86, *p* = .003, and waist circumferences, *U* = 64.0, *z = *2.83, *p* = .003, than the malnourished males. This was reflected in the overall sample with significantly lower BMIs, *U* = 2352.5, *z = *2.34, *p* = .019, smaller mid-arm circumference, *U* = 2526.5, *z = *3.22, *p* = .001, and waist circumferences, *U* = 2291.5, *z = *3.34, *p* = .001, as well as smaller calf circumferences, *U* = 2295.0, *z = *2.27, *p* = .023, in the females than the males (Table 3).

**Table 3:** Characteristics of the total sample and of the malnourished participants compared between genders using Mann-Whitney analysis and reported as median (range).

		Total sample (n = 125) (74 M, 51 F)		Malnourished participants (n = 19) (10 M, 9 F)	
Age (years)	Male	70.5 (35.0–86.0)		74.5 (66.0–81.0)	
	Female	68.0 (42.0–92.0)		72.0 (61.0–87.0)	
PD Duration (years)	Male	6.0 (0.3–31.0)		9.0 (1.0–26.0)	
	Female	6.0 (0.0–25.5)		5.0 (1.0–11.0)	
Height (cm)	Male	172.75 (156.0–186.0)*		169.5 (158.5–174.0)*	
	Female	160.0 (140.5–173.5)*		154.5 (140.5–171.5)*	
Weight (kg)	Male	75.5 (49.8–145.0)*		63.1 (49.8–100.6)*	
	Female	60.8 (40.7–128.8)*		46.7 (40.7–58.7)*	
BMI (kg/m^2^)	Male	26.1 (19.1–46.3)*		21.3 (19.5–33.2)*	
	Female	23.6 (17.0–49.5)*		19.5 (17.7–21.6)*	
MAC (cm)	Male	30.5 (23.0–41.0)*		27.6 (23.0–35.0)*	
	Female	27.0 (21.0–43.0)*		22.0 (21.0–28.0)*	
Waist circumference (cm)	Male	96.5 (74.0–149.5)*		86.75 (78.0–116.0)*	
	Female	86.5 (65.5–117.0)*		73.5 (65.5–88.0)*	
Calf circumference (cm)	Male	36.75 (28.0–48.5)*		33.75 (28.0–39.0)	
	Female	35.0 (29.0–43.0)*		32.0 (29.0–36.0)	
PG-SGA score	Male	3 (0–15)		8 (4–15)	
	Female	3 (0–13)		10 (8–13)	
		Male	Female	Male	Female
H&Y†	0	0 (0%)	2 (4%)	0 (0%)	0 (0%)
	1	17 (23%)	11 (22%)	1 (10%)	0 (0%)
	2	26 (35%)	18 (35%)	4 (40%)	3 (33%)
	3	27 (37%)	15 (29%)	3 (30%)	4 (45%)
	4	3 (4%)	5 (10%)	1 (10%)	2 (22%)
	5	1 (1%)	0 (0%)	1 (10%)	0 (0%)

* Statistically significant difference between genders (p<0.05).

† Reported as frequencies (percentages). Abbreviations: BMI = body mass index, H&Y = Hoehn & Yahr, MAC = mid-arm circumference, PD = Parkinson’s disease, PG-SGA = Patient-Generated Subjective Global Assessment.

## Discussion

The current study resulted in a malnutrition diagnosis in 15% of the sample of community-dwelling adults with Parkinson’s disease. This result is higher than previous studies where 0–2% of PWP were diagnosed with malnutrition. The discrepancy could be a result of the different assessment tools used. It could also be related to the fact that the samples in the studies by Wang, et al [1] and Barichella, et al [2], included participants that were recruited from a Movement Disorders outpatient clinic and physiotherapy participants, respectively. As a result, the results may not be generalizable to the wider community-dwelling population of PWP in which the rates of malnutrition may be higher. This may have also been the case in the present study. The most socially isolated and those with higher disease severity are also the least likely to participate in research, regardless of setting. This is reflected in the current sample where only 1 participant was classified as having a Hoehn & Yahr classification equal to 5 and only eight were classified as having a Hoehn & Yahr classification equal to 4.

Comparatively, estimates of malnutrition in community-dwelling adults without PD ranges from 3−11% with the risk of malnutrition between 15% and 38% [12], [21]–[24]. The disparity in results again arises from the differences in tool used, the age range of the samples and the sample sources. The frequency in the current sample of PWP is also higher than that reported in the general population. This is not wholly unexpected given the presence of factors in PD that increase the risk of malnutrition such as lack of appetite, altered taste and smell senses, poor eating skills and potentially higher energy expenditures. Disturbance of autonomic function of the gastrointestinal tract in PD including dysphagia, delayed gastric emptying and constipation is well-documented [25] and it is has been suggested that these disturbances, especially constipation, precede the motor symptoms [26]. The proportion of the malnourished with reduced intake due to loss of appetite, constipation, diarrhea, problems swallowing and early satiety was significantly higher than the proportion of well-nourished in the current study. The total PG-SGA score particularly reflects the presence of these nutrition impact symptoms. The 30% of the well-nourished participants with a PG-SGA score placing them in the triage categories requiring intervention are potentially at risk of becoming malnourished if those symptoms are not appropriately managed.

Significant unintentional weight loss is not uncommon in the elderly population with between 17−35% of community-dwelling older adults losing at least 5% of their baseline weight over a 3 year period [13] resulting in significantly lower BMIs when compared to non-weight losers [27]. A greater number of PWP (60%) reportedly lose weight unintentionally following diagnosis resulting in significantly lower body BMIs than those who do not lose weight [4]. Lower BMIs are also found in malnourished PWP compared to well-nourished [1]. Similarly, the current study found a higher percentage of weight losers (43%) than that reported in the general population, and BMIs were significantly lower in the malnourished group compared to the well-nourished group. The physical examination in the PG-SGA also resulted in significantly reduced fat stores and muscle status in the malnourished group in this study when compared to the well-nourished. Therefore, the current results may more accurately reflect the extent of malnutrition in PWP.

Females generally are at greater risk of unintentional weight loss and malnutrition than males [22], [28]. This appears to also be true in PWP with previous studies [1], [2] and the current study supporting this finding. The significantly larger average values for BMI and waist circumference (an indicator of adiposity [29]) coupled with the evidence of muscle loss in the PG-SGA assessment in the malnourished males may indicate that males in this group tend towards sarcopenic obesity while the females exhibit both fat and muscle loss.

This study is the largest to report the extent of malnutrition in community dwelling PWP and the first to do so in Australia. In Australia, an estimated 20% of PWP are under the age of 65 with over 2,000 aged in their 30 s and 40 s [30]. Therefore, the age range in the current sample is representative of the Australian PD population. While older age is a risk factor for malnutrition [31], Parkinson’s disease has also been reported as an independent risk factor for malnutrition [32]. Therefore it is important to understand the extent of malnutrition in PWP of any age, and there were malnourished participants in the current sample (15.8%) who were under the age of 65 years. Future studies in a larger sample could attempt to further classify the extent of malnutrition in younger PWP vs older PWP and to identify the risk factors of importance in each age group.

Another strength of this study is the use of a nutrition assessment tool applicable to any age that includes, in addition to recent weight changes and a physical examination, the presence of symptoms that are likely to influence intake in PWP. This study also highlights the fact that anthropometric measures alone would not have identified the malnourished in this population. Only 3 participants had BMI values in the underweight range (<18.5 kg/m^2^), no one had a mid-arm circumference below 21 cm, and two had calf circumferences less than 31 cm. This could be explained by the fact that a higher BMI range to detect underweight may be more appropriate in the older participants (≥65 years) [33]. However, a multi-factorial approach to the diagnosis including recent weight loss, intake, nutrition impact symptoms and a thorough assessment of muscle and fat status resulted in a higher proportion of participants classified as malnourished.

Limitations in the current study include the limited reach of the sampling process to the frailest and most vulnerable of the community-dwelling PD population. Although the current study involved data collection at participants’ homes, some potential participants declined the invitation to participate stating that they were not well enough or their Parkinson’s disease symptoms were too advanced to participate. The majority of the sample also lived within the urban area of Brisbane, Queensland, Australia where access to services and food supplies is not as limited as it might be in a more rural setting. Despite the high frequency of malnutrition, the small sample size limited the number of malnourished participants with whom comparisons could be made and potentially limits the ability to generalise the results. However, the current sample size exceeds that of other studies reporting the rate of malnutrition in community-dwelling people with Parkinson’s disease (n = 61 [2] and n = 117 [1]).

Another potential limitation in this study is the lack of validation of the available nutrition assessment tools for the Parkinson’s disease population. Therefore, the tool itself may influence the validity of the results. Worksheets 2 and 3 of the PG-SGA also did not significantly contribute to the overall score nor were they different between groups. These boxes may be of particular value in acutely ill populations, for which the tool was created and has since been validated, rather than in a chronic condition such as Parkinson’s disease. Therefore, the triage categories may not be appropriate in this population. However, the SGA is a robust nutrition assessment tool that has been widely used [16] and is considered a valid tool in community populations, regardless of disease state. Further exploration of appropriate nutrition assessment tools within this population is still warranted.

The first step to addressing malnutrition, the loss of lean body mass and the associated poor outcomes is identifying the problem. This study highlights the fact that malnutrition is prevalent in this sample of PWP. However, as with malnutrition in other populations, it remains under-recognised and undiagnosed. The health professionals who have the most contact with these individuals in the community setting should screen for malnutrition at regular intervals and provide appropriate referrals for nutrition-related care for those who are at risk of malnutrition.
